# Koopman-based linearization of preparatory EEG dynamics in Parkinson’s disease during galvanic vestibular stimulation

**DOI:** 10.3389/fnhum.2025.1566566

**Published:** 2025-05-14

**Authors:** Maryam Kia, Maryam S. Mirian, Saeed Soori, Saeed Saedi, Emad Arasteh, Mohamad Hosein Faramarzi, Abhijit Chinchani, Soojin Lee, Artur Luczak, Martin J. McKeown

**Affiliations:** ^1^Department of Neuroscience, Canadian Centre for Behavioural Neuroscience, University of Lethbridge, Lethbridge, AB, Canada; ^2^Pacific Parkinson Research Centre, University of British Columbia, Vancouver, BC, Canada; ^3^Department of Computer Science, University of Toronto, Toronto, ON, Canada; ^4^Department of Electrical and Computer Engineering, University of Tehran, Tehran, Iran; ^5^Department of Neonatology, Wilhelmina Children’s Hospital, University Medical Center Utrecht, Utrecht, Netherlands; ^6^Department of Electrical and Computer Engineering, University of Sharif, Tehran, Iran; ^7^Department of Bioinformatics, University of British Columbia, Vancouver, BC, Canada; ^8^Department of Medicine (Neurology), University of British Columbia, Vancouver, BC, Canada

**Keywords:** Parkinson’s disease, motor control, galvanic vestibular stimulation, Koopman operator theory, deep neural network

## Abstract

**Introduction:**

Parkinson’s disease (PD) impairs motor preparation due to basal ganglia dysfunction, contributing to motor deficits. Galvanic Vestibular Stimulation (GVS), a non-invasive neuromodulation technique, shows promise in enhancing motor function in PD, but its underlying neural mechanisms are poorly understood. This study employs a Deep Koopman model to linearize and analyze preparatory EEG dynamics in PD, hypothesizing that GVS restores cortical activity patterns critical for motor planning.

**Methods:**

EEG data from 18 PD participants (on/off medication) and 18 healthy controls were collected during a preparatory phase of a motor task under three conditions: sham, GVS1 (50–100 Hz multi-sine), and GVS2 (100–150 Hz multi-sine). A Deep Koopman framework mapped EEG signals into a three-dimensional latent space for linear dynamical analysis. Temporal dynamics were assessed via eigenvalue analysis, spatial contributions via regression-based scalp mapping, and motor performance correlations via Pearson’s coefficients. A Linear Quadratic Regulator (LQR) simulated control of PD dynamics toward healthy patterns.

**Results:**

The Deep Koopman model accurately captured EEG dynamics, with eigenvalue analysis showing no significant temporal dynamic differences across groups. Spatial contribution analysis revealed that PD-Off sham conditions deviated most from healthy control EEG patterns, while GVS and medication significantly reduced these deviations, aligning PD patterns closer to controls. Closer alignment correlated with improved motor performance metrics, including reduced reaction and squeeze times. LQR control effectively guided PD neural dynamics toward healthy trajectories in the latent space.

**Discussion:**

GVS enhances motor preparation in PD by restoring healthy cortical EEG patterns, with additive benefits from dopaminergic medication. The Deep Koopman framework offers a powerful approach for dissecting complex EEG dynamics and designing targeted neuromodulation strategies. These findings elucidate GVS’s therapeutic mechanisms and highlight its potential for personalized PD interventions, warranting further exploration in larger cohorts and varied stimulation protocols.

## 1 Introduction

Parkinson’s disease (PD) is a progressive neurodegenerative disorder that affects approximately 1% of individuals over the age of 60, with its prevalence increasing with age (Tysnes and Storstein, 2017). It is characterized by motor symptoms, including bradykinesia, rigidity, and tremors, as well as non-motor symptoms that include cognitive decline and autonomic dysfunction. Research has demonstrated that both motor and non-motor symptoms of PD can be modulated by external interventions. For instance, sensory stimuli like visual and auditory cues have been shown to alleviate motor and sensory impairments in PD (Muthukrishnan et al., 2019; Sacrey et al., 2011; Zadeh et al., 2024). Additionally, electrical neuromodulation, such as deep brain stimulation (DBS), have proven effective in mitigating motor symptoms and enhancing quality of life, offering a valuable complement to pharmacological treatments (Lachance et al., 2018). However, despite the potential of these approaches, their utility is often constrained by several factors, including variability in patient response, limited accessibility, and diminishing efficacy as the disease progresses. Further investigation into the mechanisms underlying PD’s responsiveness to external stimuli is a key step toward overcoming these challenges and the exploration of innovative therapeutic strategies. Among the current effective methods, galvanic vestibular stimulation (GVS), a non-invasive brain stimulation technique, shows significant promise and merits thorough evaluation as a potentially effective and accessible intervention. GVS applies low-intensity electrical currents via electrodes placed behind the ears to stimulate the vestibular system (Pires et al., 2022). Studies have shown that GVS can enhance balance and motor control in PD patients, offering a means of modulating motor symptoms (Khoshnam et al., 2018; Lee et al., 2021). Previous work within our group has extensively examined the behavioral effects of GVS in PD, demonstrating its potential to enhance motor performance. Findings indicate that GVS improves reaction times, reduces bradykinesia, and enhances movement precision in visuomotor and motor execution tasks. In visuomotor tracking experiments, noisy GVS led to faster responses, smoother trajectory control, and improved adaptation to task demands, particularly in more challenging conditions. Additionally, motor execution tasks showed that high-frequency GVS facilitated movement initiation and increased response vigor, with effects varying based on stimulation parameters and individual motor deficits (Lee, 2019). Despite these promising outcomes, the underlying mechanisms by which GVS influences motor function, particularly in the context of motor control, remain poorly understood.

The effects of GVS on motor performance in PD are likely multifaceted, as motor control involves the integration of sensory information with motor commands to plan, initiate, and execute movements (Gallivan et al., 2018; Shadmehr et al., 2010). While certain aspects of motor control, such as well-learned movement patterns, remain stable, the neural populations involved in planning and executing movements are highly dynamic, particularly during the preparatory phase preceding movement execution. This phase reflects neural activity that anticipates movement, laying the groundwork for effective motor initiation and coordination (Churchland et al., 2010; Churchland et al., 2012).

Exploring the electroencephalogram (EEG) dynamics during the motor preparation phase in individuals with Parkinson’s disease (PD) is especially relevant given the critical role of dopamine in this process. Dopamine modulates readiness potentials (Köhler et al., 2024), facilitates gradual firing increases in motor-related regions (Alm, 2021), and enhances cortico-striatal interactions (Michely et al., 2015), all of which are essential for efficient motor preparation and initiation. Moreover, dopamine’s influence on synaptic plasticity, particularly long-term potentiation (LTP) in the motor cortex (Molina-Luna et al., 2009), underscores its importance in motor skill learning and preparatory states. Examining EEG changes during motor preparation can reveal how dopamine-mediated oscillatory activity and connectivity patterns are disrupted in PD and how treatments, such as dopaminergic medication or non-invasive brain stimulation like GVS, may ameliorate these deficits. Such insights are crucial for advancing therapeutic strategies to restore normal motor network dynamics.

From a dynamical systems perspective, motor control and preparatory activity can be conceptualized as trajectories within a high-dimensional state space, where the system transitions through preparatory states to achieve desired motor outcomes (Shenoy et al., 2011). This framework offers a powerful approach for understanding how external interventions, such as GVS, influence neural dynamics and motor behavior.

In this study, we hypothesize that GVS modulates the temporal properties of neural dynamics and/or restores and enhances activity in underactive brain regions in PD, thereby improving preparatory motor activity and motor function. Traditional approaches to studying neural dynamics often rely on non-linear methods, which are computationally intensive and challenging to generalize. To test our hypothesis, we analyzed EEG recordings during the preparatory phase of a motor task using a Deep Koopman model. Koopman operator theory offers a novel approach to analyzing non-linear systems by transforming their dynamics into a higher-dimensional space of observables, where the system evolves linearly (Koopman, 1931). While the Koopman framework has seen widespread application in engineering domains (Berger et al., 2015; Kaiser et al., 2020; Korda and Mezić, 2018), its use in neuroscience—particularly in clinical contexts like PD—remains underexplored. Recent studies have demonstrated the utility of Koopman-based methods in modeling complex brain dynamics across various applications. For instance, one study (Hellar et al., 2022) employed an embedded dynamic mode decomposition (EmDMD) technique based on Koopman theory to transform the non-linear spatiotemporal dynamics of EEG signals into a linearized spectral representation. This approach enabled the extraction of novel features, which were then used to classify EEG segments into preictal and interictal states, underscoring the potential of Koopman-based techniques for clinical EEG analysis. Similarly, another study (Li and Guo, 2024) addressed the challenge of detecting and predicting Freezing of Gait (FOG) in PD using EEG data. The researchers applied a novel DMD-ACSP (Dynamic Mode Decomposition with Analytic Common Spatial Pattern) method, which significantly outperformed traditional techniques like Fourier Transform, Wavelet Transform, and convolutional neural networks (CNNs) in FOG detection and prediction tasks. By integrating dynamic mode decomposition with spatial filtering, the study provided a robust framework for analyzing brain dynamics, paving the way for personalized neuromodulation strategies to manage FOG. These advancements highlight the promise of Koopman-based approaches for advancing the analysis of EEG dynamics in PD and other neurological disorders.

Here we utilize a deep neural network (DNN) to map non-linear EEG data into a latent space where linear dynamical systems can be applied. This approach combines the representational power of DNNs with dynamical system modeling to achieve both linearization and dimensionality reduction of complex EEG signals, enabling predictive modeling and control strategies within a linear latent space. Group-specific models were trained, and the EEG data were decomposed into a three-dimensional Koopman subspace. These latent dynamics were further analyzed using eigenvalue analysis, spatial contribution analysis, and a Linear Quadratic Regulator (LQR) control strategy. This innovative methodology provides a powerful framework for investigating GVS-induced neural dynamics and offers practical insights for designing targeted interventions to enhance motor function in Parkinson’s disease.

## 2 Materials and methods

**FIGURE 1:**
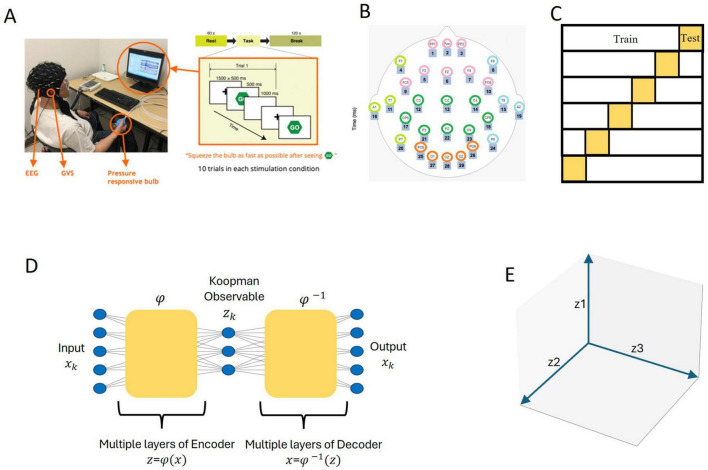
Schematic representation of the methodological workflow. **(A)** Data acquisition: the setup included electroencephalogram (EEG) recording during a visuomotor task. Participants completed 10 trials for each stimulation condition (Sham, GVS1, and GVS2). **(B)** EEG data preprocessing: signals from 27 electrodes were grouped into five anatomically defined brain regions for analysis. **(C)** A total of 6-fold cross-validation: the dataset was partitioned into training and test subsets to evaluate model performance within a 6-fold cross-validation framework. **(D)** Deep koopman framework: an autoencoder was used to map the five-dimensional EEG input into a three-dimensional Koopman latent space, where the Koopman operator acts linearly to facilitate analysis. **(E)** Koopman-based analysis: the three-dimensional Koopman latent space representation was leveraged to examine temporal dynamics (via eigenvalue analysis) and spatial contributions (via scalp mapping), enabling direct comparisons across experimental groups and stimulation conditions.

Figure 1 provides an overview of the methodological steps, including data acquisition, the visuo-motor task, data preprocessing, and model architecture. Each step is depicted in the diagram, with additional details provided in the figure caption and the subsequent subsections.

### 2.1 Data description

#### 2.1.1 Ethics statement

The study protocol was approved by the Clinical Research Ethics Board at the University of British Columbia (UBC), and the recruitment was conducted at the Pacific Parkinson’s Research Centre (PPRC) at UBC. All participants gave written, informed consent before participation.

#### 2.1.2 Participants, Behavioral tasks and EEG data acquisition

Electroencephalogram and behavioral data used in this study were obtained from prior study (Lee, 2019), including 20 participants with PD and 22 HC. All PD participants had a clinical diagnosis of idiopathic Parkinson’s disease and were in Hoehn and Yahr stages 1–3, indicating mild to moderate disease severity. Individuals with atypical parkinsonism, advanced PD, or major neurological or psychiatric comorbidities, were excluded. Data collection was conducted under two conditions: “off medication” (PD-Off), following a 12 h overnight withdrawal, and “on medication” (PD-On), after participants had taken their regular dose of Levadopa. No severe side effects of Levodopa were reported or observed during the sessions. Participants selected for the experiment were all able to perform the hand-squeeze task used in the study. EEG recordings were obtained from 27 scalp electrodes positioned according to the international 10–20 system, with a sampling rate of 1 kHz.

The experiment employed a block design, with each block corresponding to one of three stimulation conditions: sham stimulation (no stimulation) or GVS using two distinct waveforms, GVS1 (50–100 Hz multi-sine) and GVS2 (100–150 Hz multi-sine). Participants sat in front of a computer screen and were instructed to respond as quickly as possible to a visual “Go” cue by squeezing a rubber bulb. Each block consisted of 10 trials, and each trial followed a fixed sequence: a fixation screen displayed for 1,500 ± 500 ms, a “Go” signal presented for 500 ms, and a blank screen lasting 1,000 ms. Various motor performance measures were recorded during each trial. Further experimental and behavioral details are provided in (Lee, 2019).

#### 2.1.3 Data preprocessing

From the original dataset, two PD participants were excluded due to excessive EEG noise. To maintain balance and minimize potential group-size bias, we selected the 18 HC participants with the highest EEG data quality from the original pool of 22. This ensured a matched sample design, which was critical for methodological consistency and statistical validity, especially in the K-fold cross-validation framework used for model training and evaluation.

The 1,000 ms preparatory interval preceding the “Go” signal was extracted from each trial to analyze motor preparation dynamics. At 1 kHz sampling, this yielded 1,000 EEG samples per trial. With 10 trials per subject, each participant contributed 10,000 samples, resulting in 180,000 samples per group (18 subjects × 10 trials × 1,000 samples).

The 27 scalp electrodes were grouped into five anatomically defined regions of interest (ROIs)—frontal, central, parietal, occipital, and temporal—to facilitate analysis. This grouping method was selected to preserve regionally specific cortical activity relevant to motor preparation, minimizing variability across individual electrodes while reducing redundancy from volume conduction. Since adjacent electrodes often capture overlapping neural activity, averaging within ROIs enhances interpretability and ensures that spatial contributions reflect broader cortical patterns rather than isolated electrode-level signals. From a computational perspective, reducing the input to five regionally averaged signals maintained a computationally efficient representation while preserving essential EEG features.

For analysis, the data were divided into seven experimental conditions representing participant type, medication status, and stimulation condition: HC-Sham, PD-Off-Sham, PD-On-Sham, PD-Off-GVS1, PD-On-GVS1, PD-Off-GVS2, and PD-On-GVS2. Each group was analyzed independently to assess the effects of medication and stimulation on brain activity and motor performance.

### 2.2 Koopman model

#### 2.2.1 Koopman operator theory

Consider a discrete-time dynamical system:


$$x_{k+1}=F\,\left(x_{k}\right),x_{k}\in M\subseteq R^{n}$$

Here, *x_k_* is an n-dimensional state vector at time step k, and *M* ⊆ *R^n^* denotes the state-space (or manifold) in which the system evolves. The map *F* : *M* → *M* defines the non-linear update rule for the system.

We call any function *g* : *M* → *R* an observable of the system, meaning that it maps states *x* ∈ *M* to real values. The set of all such observables forms a (potentially) infinite-dimensional vector space, as infinitely many functions can be constructed on the state space (e.g., polynomials, exponentials, neural-network-based features). This infinite-dimensional property allows the Koopman operator to represent complex, non-linear dynamics through linear evolution in a higher-dimensional functional space.

The Koopman operator, denoted K, is a linear operator acting on this space of observables. Specifically, for any observable g, its action is defined as:


$$K.g\,\left(x_{k}\right)=g\,\left(F\,\left(x_{k}\right)\right)=g\,\left(x_{k+1}\right)$$

Thus, K advances the value of g by one time step.

For a finite number of observable functions, *K* can be approximated as a finite-dimensional operator, enabling numerical analysis of the system’s dynamics. We refer the reader to Budisić et al. (2012) for a more detailed discussion of the Koopman operator basics.

A key challenge in practical applications of the Koopman method is selecting appropriate observable functions. Dynamic Mode Decomposition (DMD) has traditionally been used to approximate the Koopman operator (Schmid, 2010; Williams et al., 2015), but it relies on linear measurements of the system, which are often insufficient to span a Koopman subspace in non-linear systems.

In contrast, deep learning methods offer a data-driven approach for identifying observables (Morton et al., 2019; Takeishi et al., 2017). In the DNN approach, the observables are learned to approximate the Koopman operator, forming a finite-dimensional linear representation of the system. This representation satisfies:


$$\varphi \,\left(x_{k+1}\right)=K.\varphi \,\left(x_{k}\right)=\lambda .\varphi \,\left(x_{k}\right)$$

Where φ(*x*_*k*_) represents the learned observables, and K is the Koopman operator in the latent space. This formulation transforms the non-linear update rule into a standard linear difference equation:


$$z_{k+1}=K\,z_{k}.$$

Where z_k_ = *φ* (x_k_) denotes the transformed state in the Koopman latent space.

The eigenvalues λ of the Koopman operator characterize critical system properties, including stability and dynamic behavior (Korda et al., 2020; Mezic, 2017):

• Stability: A system is considered stable if all eigenvalues satisfy |λ| < 1
• Non-Oscillatory Dynamics: A real eigenvalue describes exponential damping without oscillations.
• Oscillatory Behavior: Complex conjugate eigenvalues indicate oscillatory dynamics with exponential damping.

This Koopman-based framework enables the linearization of non-linear dynamics, allowing for system analysis, prediction, and control within a finite-dimensional space.

#### 2.2.2 Deep neural network for Koopman operator

To implement the Koopman method, we utilized the DNN framework introduced by Lusch et al. (2017), which employs a deep autoencoder to learn a finite-dimensional representation and approximate the Koopman operator.

**FIGURE 2:**
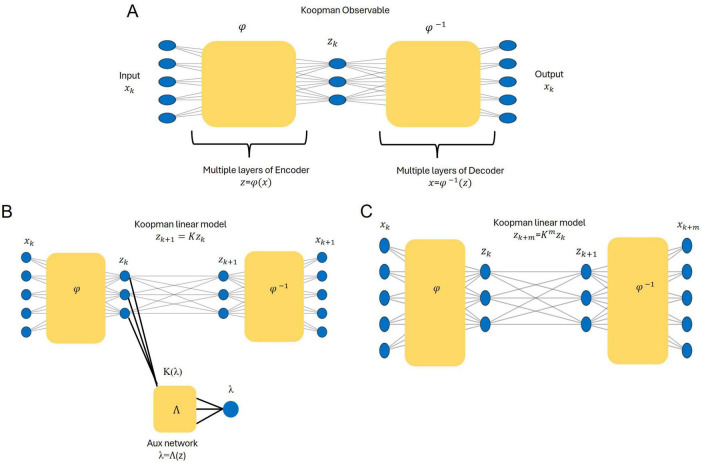
Architecture and workflow of the deep Koopman framework adapted from Lusch et al. (2017). **(A)** The deep autoencoder maps input states (*x*_*k*_) into a three-dimensional Koopman latent space using the encoder(φ), while the observables (z_k_) are reconstructed back to the original states via the decoder (φ^−**1**^). **(B)** An auxiliary network is employed to identify the eigenvalue spectrum and parametrize the Koopman operator, enabling the linear propagation of observables **z**_**k** + **1**_. **(C)** Multi-step predictions are achieved by propagating observables over time *z*_*k*+*m*_ = *K^m^z_k_*, with the decoder reconstructing the predicted states ${\hat{\text{x}}}_{k+m}=\varphi^{-1}\,\left(K^{m}\,\varphi \,\left(x_{k}\right)\right)$.

Figure 2A depicts the core network architecture for identifying the Koopman model. In our framework, *φ* represents the encoder network in the deep autoencoder, mapping the raw EEG states *x* ∈ *R*^5^ into a three-dimensional Koopman latent space *z* ∈ *R*^3^. Formally, *φ* is a multi-layer neural network of the form


$$z=\varphi \,\left(x\right),z\in R^{3}$$

Each layer applies an affine transformation (weights and biases) followed by a non-linear activation function. Unlike predefined analytic functions, φ is learned directly from data, with its final form determined by the network’s trained parameters. This learned transformation ensures that the Koopman representation remains expressive while maintaining interpretability and computational efficiency. The bottleneck layer of the autoencoder defines the latent space, which approximates the observables and provides the foundation for linearizing the system’s dynamics. Once encoded, the system evolution in this latent space follows linear dynamics under the Koopman operator. The decoder network φ^−1^ reconstructs the original EEG dynamics from the latent representation, ensuring that the transformation preserves essential temporal patterns while enabling a data-driven Koopman approximation.

An auxiliary network refines the Koopman model by extracting eigenvalues from the latent representation. The auxiliary network takes the encoded latent representations [*z* = (*φ*(*x*))] as input and outputs the eigenvalue parameters of the Koopman matrix (*K*). It maps real eigenvalues directly and computes complex-conjugate pairs using estimated parameters (μ, ω), defining the corresponding block structure of K (Figure 2B). In the latent space, the Koopman operator enables linear propagation of the system’s dynamics across future time steps. Figure 2C demonstrates this process: the predicted observables in the latent space are decoded to reconstruct the future states of the original system. The multi-step prediction process is mathematically described as:


$$z_{k}=\varphi \,\left(x_{k}\right)$$

$$z_{k+1}=K\,z_{k}$$

…

$$z_{k+m}=K^{m}\,z_{k}$$

$${\hat{\text{x}}}_{\text{k}+\text{m}}=\varphi^{-1}\,\left(K^{m}\,\varphi \,\left(x_{k}\right)\right)$$

Details of the loss functions used to train the autoencoder, including reconstruction loss, linearity loss, and prediction loss, are provided in the Supplementary materials.

##### 2.2.2.1 Training and parameters setting

The autoencoder was trained using the five-dimensional EEG time series as input. In this DNN framework, which approximates the Koopman operator in a finite-dimensional setting, the dimensionality of the observables (i.e., the latent space) must be predefined based on prior knowledge of the system. In our approach, the dimensionality of the latent space corresponds to the number of eigenvalues in the Koopman matrix (*K*). Based on previous research (Yang et al., 2021), to enhance the linear modeling of neural activity, we chose our model to incorporate both damping and oscillatory dynamics, as captured by real and complex-conjugate eigenvalues, respectively. From the available odd-dimensional options for the latent space we chose a three-dimensional space to balance model interpretability, computational efficiency, and the ability to capture essential neural dynamics. The bottleneck layer constrains the Koopman representation to three dimensions, ensuring that the learned dynamics remain low-dimensional while preserving key state transitions. This dimensionality selection remains flexible and can be extended to higher-dimensional representations if necessary for more complex dynamical structures.

Hyperparameter tuning for each group’s network was conducted using a grid search in the parameter space, following the methodology described in Goodfellow et al. (2016). Full details of the network architecture and hyperparameters for each model are provided in Supplementary Table 1.

To ensure robust model evaluation and stable convergence, we employed a 6-fold cross-validation approach. The time-series data from each experimental group (18 subjects per group) were divided into six folds, with 15 subjects used for training and three subjects held out for testing in each iteration. This procedure was repeated for each group to optimize model performance and assess generalizability.

Training stability was assessed by monitoring a combined reconstruction/prediction/linearity loss, ensuring it consistently decreased over epochs.

##### 2.2.2.2 Model evaluation

The performance of the deep Koopman model was evaluated to assess its effectiveness in reconstructing neural dynamics and predicting future states across the seven experimental groups. Separate networks were trained for each group using their respective training sets, and performance was evaluated on the test sets to ensure generalizability to unseen data. The primary evaluation metric was the network error, defined as the total loss function used during the autoencoder training process. Model performance was further assessed based on two key criteria: the accuracy of signal reconstruction from the low-dimensional latent dynamics and the ability to predict future dynamics. This approach provided a comprehensive evaluation of the model’s capability to capture and generalize neural activity across conditions.

Forward prediction performance was assessed by calculating multi-step prediction errors. We defined m-step ahead prediction as the estimation of the original dynamics (*x_k_*) using values from up to m-time steps earlier, denoted as ${\hat{x}}_{k|k-m}$. For m = 1–15, the normalized mean squared error was calculated for each step as:


$$N\,M\,S\,E=\frac{\frac{1}{\text{n}}\,\sum_{1}^{\text{n}}{\left({\text{x}}_{\text{k}}-{\hat{\text{x}}}_{\text{k}}\right)}^{2}}{{\text{x}}_{\text{mean}}}$$

Where *n* is the total number of samples in the test set and x_mean_ is the mean value of the original signal.

### 2.3 Koopman latent representation

The proposed method transforms the five-dimensional EEG time series into a three-dimensional Koopman latent space while simultaneously constructing a linear dynamical model. This transformation provides a reduced-dimensional representation that encapsulates the system’s temporal evolution in a linearized state space. The Koopman framework facilitates the tracking of trajectories within this latent space, enabling detailed insights into the system’s temporal and spatial dynamics. The three-dimensional Koopman representation and its corresponding linear model were utilized to compare neural dynamics across experimental groups. Specifically, the Koopman transformation was assessed for its ability to:

1- Capture temporal dynamics: Eigenvalue analysis of the linear Koopman operator was performed to evaluate the stability and oscillatory properties of the system.
2- Analyze spatial contributions: Regression-based back-projections were used to examine how EEG activity from different brain regions contributed to the latent dynamics.

#### 2.3.1 Eigenvalue analysis

Eigenvalue analysis was conducted to characterize the temporal dynamics represented in the Koopman latent space for each experimental group. The models consistently identified three eigenvalues: one real eigenvalue, reflecting non-oscillatory dynamics with exponential damping, and a pair of complex-conjugate eigenvalues, representing oscillatory activity. Systematic comparisons of these eigenvalues across experimental groups were performed to uncover differences in temporal evolution and to examine the effects of medication and stimulation conditions on the underlying neural dynamics.

#### 2.3.2 Spatial contribution

A linear regression model was used to map the three-dimensional Koopman latent representations back onto the EEG scalp space. This mapping produced regression coefficients that quantified the relative contribution of each EEG electrode to the latent dynamics. Since each experimental group was modeled using an independently trained Koopman network, the latent space representations exist in relative coordinate systems unique to each model rather than a shared absolute reference frame. Consequently, direct comparisons of latent feature positions across models are not meaningful, as coordinate system differences could confound interpretations. To address this limitation, we anchored comparisons in the EEG observational space, where the spatial contribution of EEG electrodes remains consistent across all groups.

To assess differences in these spatial contribution patterns across experimental groups, pairwise Euclidean distances were calculated between the coefficients of each experimental group and those of the HC group, which served as a baseline reference for optimal brain function. For each latent dimension (*z*_*i*_, i = 1,2,3). The Euclidean distance *d*_*i*_ was computed as:


$$d_{i}=\sqrt{\sum_{j=1}^{n}{\left(\left|w_{i\,j}^{P\,D-o\,n}\right|-\left|w_{i\,j}^{H\,C}\right|\right)}^{2}}$$

where $\left|w_{i\,j}^{P\,D-o\,n}\right|\,a\,n\,d\,\left|w_{i\,j}^{H\,C}\right|$ are the absolute values of the regression coefficients for the j-th EEG electrode in the PD-On group and HC group, respectively, and n is the total number of electrodes.

##### 2.3.2.1 Association with motor behavior

We investigated the relationship between spatial activations and behavioral metrics across experimental groups to determine whether the Koopman latent dynamics derived from preparatory EEG activity correlated with motor behavior. Spatial activations were quantified as the mean deviations from the HC baseline for each experimental group. At the same time, behavioral performance was characterized using four metrics: Movement Time, Squeeze Time, Reaction Time, and Peak Time, averaged across trials for each group. Pearson’s correlation coefficient (CC) was calculated for each Koopman latent dimension (*z*_1_, *z*_2_, *z*_3_) to evaluate the association between spatial activations and behavioral metrics. This group-level analysis provided valuable insights into the relationship between preparatory EEG dynamics and motor behavior under different experimental conditions.

#### 2.3.3 LQR control for the linearized neural dynamics

Once the Koopman operator K has been identified, we formulate an LQR to control the system in its latent space. Since the Koopman-identified system is autonomous (i.e., it lacks explicit input dynamics), we introduce a control input by defining an identity matrix *B* in the latent space:


$$z_{k+1}=K\,z_{k}+B\,u_{k}$$

where

• *z_k_* is the state in the Koopman latent space.
• K is the Koopman operator governing state evolution.
• B is an identity matrix allowing actuation in all latent dimensions.
• *u_k_* is the control input applied in the latent space.

To ensure that the system is controllable, we check that the controllability matrix:


$$C=\left[B,K\,B,K^{2}\,B,\ldots ,K^{n-1}\,B\right]$$

has full rank, ensuring that all Koopman latent states can be influenced by control inputs. If *C* has full row rank, then the system is fully controllable, meaning LQR can be effectively applied.

Instead of stabilizing the system around an equilibrium, we design LQR for trajectory tracking, where the control law minimizes the deviation from a desired reference trajectory *r_k_*. The cost function is defined as:


$$J=\sum_{0}^{N}\left({\left(z_{k}-r_{k}\right)}^{T}\,Q\,\left(z_{k}-r_{k}\right)+u_{k}^{T}\,R\,u_{k}\right)$$

where:

• *r_k_* is the desired reference trajectory in the latent space.
• *Q* penalizes deviations from the desired trajectory *r_k_*.
• *R* penalizes excessive control effort.

The optimal LQR control law for tracking is:


$$u_{k}=-K_{L\,Q\,R}\,\left(z_{k}-r_{k}\right)$$

where *K*_*LQR*_ the LQR gain matrix calculated by solving the discrete-time algebraic Riccati equation (DARE). Substituting this into the system dynamics, the closed-loop system becomes:


$$z_{k+1}=\left(K-B\,K_{L\,Q\,R}\,z_{k}\right)+B\,K_{L\,Q\,R}\,r_{k}$$

where:

• The matrix *K^cl^* = *K*−*BK*_*LQR*_*z*_*k*_ defines the controlled system dynamics.
• The eigenvalues of *K^cl^* determine the stability of the controlled system.

This formulation ensures that the control input minimizes trajectory deviations while balancing stability and control effort.

To apply this control strategy, we selected the neural dynamics of the HC group as the reference trajectory, while the system to be controlled corresponded to the neural dynamics of the PD-On condition. The optimal control inputs were computed in the Koopman latent space and subsequently transformed back to the original state space, influencing the system’s evolution in the physiological domain.

### 2.4 Statistical analysis

Statistical analyses were performed using analysis of variance (ANOVA) to assess differences across experimental groups. Prior to conducting ANOVAs, the Kolmogorov-Smirnov (K-S) test was used to verify normality assumptions for each group. All *p*-values exceeded 0.05, supporting the use of ANOVA. A single-factor ANOVA was used to compare disease states (HC, PD-Off, PD-On) under sham stimulation. For comparisons involving stimulation, a two-factor ANOVA was conducted with stimulation condition (Sham, GVS1, GVS2) and medication status (PD-Off, PD-On) as independent variables. When significant main effects or interactions were found, Bonferroni-corrected post-hoc *t*-tests were applied. Full K-S test results are provided in Supplementary Tables 19, 20.

## 3 Results

### 3.1 Model performance

The performance of the deep-Koopman model was evaluated across seven experimental groups: HC-Sham, PD-Off-Sham, PD-On-Sham, PD-Off-GVS1, PD-On-GVS1, PD-Off-GVS2, PD-On-GVS2.

**FIGURE 3:**
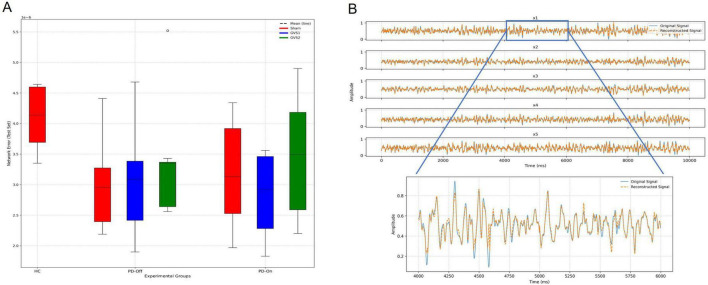
Evaluation of the deep-Koopman model performance. **(A)** Test set network errors across experimental groups (HC, PD-Off, PD-On) and stimulation conditions (Sham, GVS1, GVS2) are shown in panel **(A)**. Box plots display the distribution of test errors, with dashed lines indicating the mean. **(B)** An example of EEG reconstruction for one subject illustrates the original (blue) and reconstructed (orange) signals across all five input dimensions (x1–x5). A zoomed-in view highlights the reconstruction accuracy of the x1 signal over a 2 s interval.

Network error served as the primary evaluation metric, with detailed results for all fitted models provided in Supplementary Table 2. A comparison of average network error between groups, shown in Figure 3A, revealed higher errors for the HC group. A single-factor ANOVA for the Sham condition identified a significant difference in network test error among groups [F (2,15) = 3.8, *p* = 0.046]. However, *post-hoc* analyses with corrections for multiple comparisons did not reveal any significant pairwise differences. Comprehensive results of the ANOVA and post-hoc analyses are detailed in Supplementary Tables 3–5. Figure 3B provides an example of EEG reconstruction for one subject, showcasing the network’s capacity to accurately capture neural dynamics. The reconstruction demonstrates strong agreement between the actual and reconstructed signals across all five input dimensions, highlighting the model’s effectiveness in preserving essential neural features.

**FIGURE 4:**
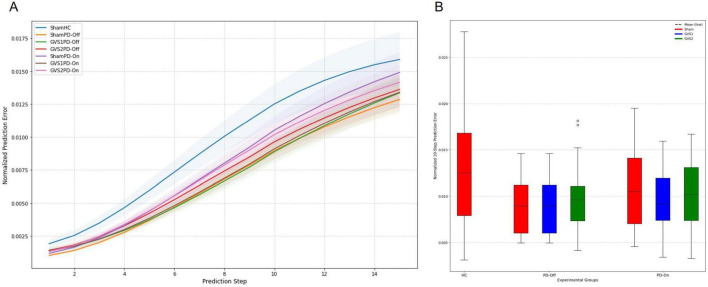
Forward prediction performance of the Deep Koopman model. **(A)** The multi-step normalized prediction error as a function of the prediction horizon (up to 15 steps) is shown for all experimental groups (HC, PD-Off, PD-On) and stimulation conditions (Sham, GVS1, GVS2). Solid lines represent the mean normalized prediction error, and shaded regions depict the standard error of the mean (SEM). **(B)** A box plot comparison of normalized 10-step prediction errors across all experimental groups and stimulation conditions illustrates the distribution of prediction errors. Dashed lines within the box plots indicate the mean prediction error for each group and condition.

The forward prediction performance of the deep Koopman model was assessed by calculating multi-step prediction errors for all groups. Figure 4A depicts the progression of prediction error as a function of the prediction horizon, demonstrating an increase in errors with longer prediction steps. Figure 4B compares normalized 10-step prediction errors across experimental groups. Across all groups, the NMSE for 10-step prediction remained below 0.02, with most conditions achieving NMSE values < 0.01. This indicates strong predictive performance. Although the HC group exhibited the highest prediction error, ANOVA of the normalized 10-step prediction errors found no significant differences between groups, suggesting comparable prediction performance across all models. Full ANOVA results are provided in Supplementary Tables 6, 7.

#### 3.1.1 Model performance on individual trials

To further evaluate model performance, trial-level analysis was conducted by examining reconstruction errors and 10-step prediction errors across all trials for each subject within each group. Statistical analysis showed no significant differences in either reconstruction errors or 10-step prediction errors across the 10 trials within any group. Comprehensive results are presented in Supplementary Table 8.

### 3.2 Eigenvalue analysis

To characterize the neural dynamics captured by the Koopman model, we analyzed the real and complex eigenvalues of the identified system for each experimental condition. The eigenvalues provide insights into neural stability and oscillatory behavior in the latent space, with differences observed between PD-On and PD-Off conditions.

• Real Eigenvalues (*λ* = *μ*): These indicate the stability of neural activity. Smaller real values (closer to 0) suggest greater stability, while values approaching 1 indicate persistent neural fluctuations.
  ◦ In PD-On, real eigenvalues were shifted slightly toward 0, suggesting increased neural stability compared to PD-Off.
  ◦ In PD-Off, real eigenvalues were closer to 1, indicating reduced stability and more persistent neural fluctuations.
• Complex Eigenvalues (*λ* = *μ* ± *iw*): These represent oscillatory activity in the latent space.
  ◦ Real Part (*μ*): As with real eigenvalues, smaller values indicate greater stability, meaning oscillations decay more effectively.
  ◦ Imaginary Part (*w*): This component determines oscillation frequency, with larger |*w*| values reflecting faster oscillations.
  ◦ In PD-On, eigenvalues indicated controlled oscillatory activity, suggesting more regulated neural behaviour.
  ◦ In PD-Off, eigenvalues had higher |*w*| values and real parts closer to 1, suggesting more persistent oscillatory activity and reduced stability.

**FIGURE 5:**
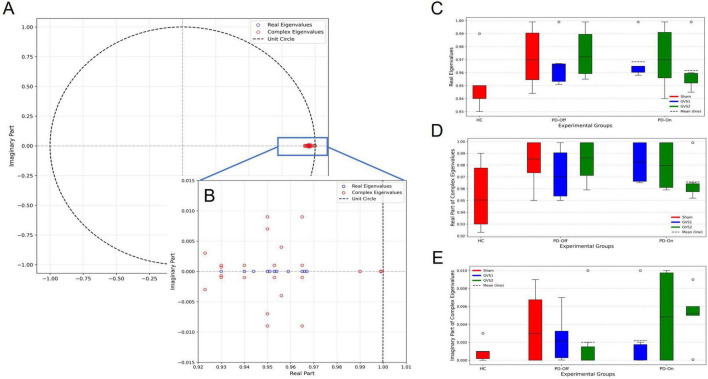
**(A)** Eigenvalues from all trained Koopman models, including one real eigenvalue and one complex-conjugate pair per model, are plotted on the unit circle, confirming model stability. **(B)** A magnified view highlights eigenvalues near the stability boundary. **(C–E)** Box plot comparisons of eigenvalue components across experimental groups (HC, PD-Off, PD-On) and stimulation conditions (Sham, GVS1, GVS2): **(C)** real eigenvalues, **(D)** real parts of complex eigenvalues, and **(E)** imaginary parts of complex eigenvalues.

Figure 5A shows the eigenvalue distributions across conditions, with PD-Off states exhibiting reduced stability and stronger oscillatory activity compared to PD-On. These shifts align with expected medication effects on motor-preparatory neural dynamics.

Statistical comparisons of eigenvalue components across conditions showed no significant differences in ANOVA; indicating that the temporal dynamics of neural activity were consistent across experimental conditions. Supplementary Tables 9–15 provide full statistical details.

### 3.3 Analysis of spatial contributions across experimental conditions

**FIGURE 6:**
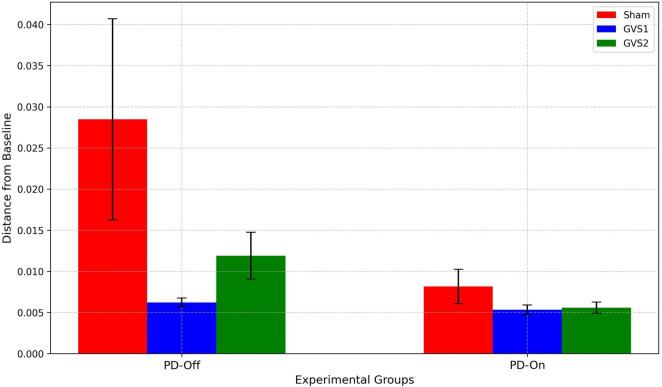
Bar plots display the average pairwise Euclidean distances of regression coefficients for Sham (red), GVS1 (blue), and GVS2 (green) groups under PD-Off and PD-On conditions. Error bars represent the standard error of the mean. Greater distances indicate larger deviations from the HC baseline, whereas smaller distances reflect closer alignment with the HC spatial contributions.

Differences in spatial contributions were evaluated by calculating pairwise Euclidean distances between the regression coefficients of each experimental group and the HC baseline, which served as a reference for optimal motor-preparatory brain function. Significant differences were observed in EEG electrode contributions, particularly in latent dimensions *z_1_*. Figure 6 presents the mean Euclidean distances from the HC baseline for each experimental group under PD-Off and PD-On conditions and across stimulation settings (Sham, GVS1, GVS2).

Under PD-Off conditions, the Sham group exhibited the largest deviation from the HC baseline, with a mean distance of 0.028 (± 0.012). In contrast, the GVS1 and GVS2 groups demonstrated reduced deviations, with mean distances of 0.0006 (± < 0.001) and 0.011 (± 0.002), respectively, indicating closer alignment with the HC spatial contributions. Under PD-On conditions, all groups showed smaller deviations from the HC baseline compared to PD-Off. The Sham group had a mean distance of 0.008 (± 0.002), while the GVS1 and GVS2 groups displayed further reductions, with mean distances of 0.005 (± < 0.001) for both conditions.

A two-factor ANOVA was conducted to assess the effects of medication status (PD-Off vs. PD-On) and stimulation condition (Sham, GVS1, GVS2) on the Euclidean distances. The results revealed a significant main effect of medication [F (1,30) = 4.65, *p* = 0.039F], indicating substantial differences in spatial contributions between PD-Off and PD-On conditions. The main effect of the stimulation condition reached moderate significance [F (2,30) = 3.17, *p* = 0.05). These findings suggest that medication significantly influences the spatial contributions of brain regions, and stimulation protocols (particularly GVS1 and GVS2) have the potential to align spatial contributions closer to the HC baseline. Detailed ANOVA results are provided in Supplementary Tables 16–18.

#### 3.3.1 Association with motor behavior

The correlation analysis identified significant associations between spatial activations derived from the Koopman latent dynamics and behavioral metrics across experimental groups. Table 1 presents the Pearson correlation coefficients (CC) and their corresponding *p*-values for each latent dimension (*z*_1_, *z*_2_, *z*_3_) and behavioral metric.

**TABLE 1:** Pearson correlation coefficients (CC) and *p*-values for the association between spatial activations and behavioral metrics across Koopman latent dimensions.

Latent dimension	Movement time	Squeeze time	Reaction time	Peak time
*z_1_*	0.62 (*p* = 0.19)	**0.96 (*p* < 0.001)**	**0.86 (*p* = 0.03)**	**0.96 (*p* < 0.001)**
*z_2_*	0.73 (*p* = 0.1)	0.62 (*p* = 0.19)	**0.87 (*p* = 0.02)**	**0.87 (*p* = 0.07)**
*z_3_*	0.61 (*p* = 0.2)	**0.87 (*p* = 0.02)**	**0.84 (*p* = 0.04)**	**0.91 (*p* = 0.01)**

Significant correlations (*p* < 0.05) are highlighted in bold.

• Latent dimension *z_1_*: Significant correlations were found for Squeeze Time (r = 0.96, *p* < 0.001), Reaction Time (r = 0.86, *p* = 0.03), and Peak Time (r = 0.96, *p* < 0.001), indicating that spatial activations in this dimension are closely related to motor behavior metrics involving timing and task duration.
• Latent dimension *z_2_*: Moderate to strong correlations were noted with Reaction Time (r = 0.87, *p* = 0.02).
• Latent dimension *z_3_*: significant correlations were observed for Squeeze Time (r = 0.87, *p* = 0.02), Reaction Time (r = 0.84, *p* = 0.04), and Peak Time (r = 0.91, *p* = 0.01), highlighting this dimension’s role in capturing motor behaviors related to fine motor control and task completion timing.

Notably, spatial activations showed stronger and more consistent correlations with Reaction Time, and Peak Time measure than with Movement Time. These differences in *p*-values reflect variability in how reliably each behavioral metric tracked changes in latent EEG dynamics across groups. Movement Time and the Squeeze Time metric exhibited higher inter-subject variability, resulting in weaker correlations and higher *p*-values.

**FIGURE 7:**
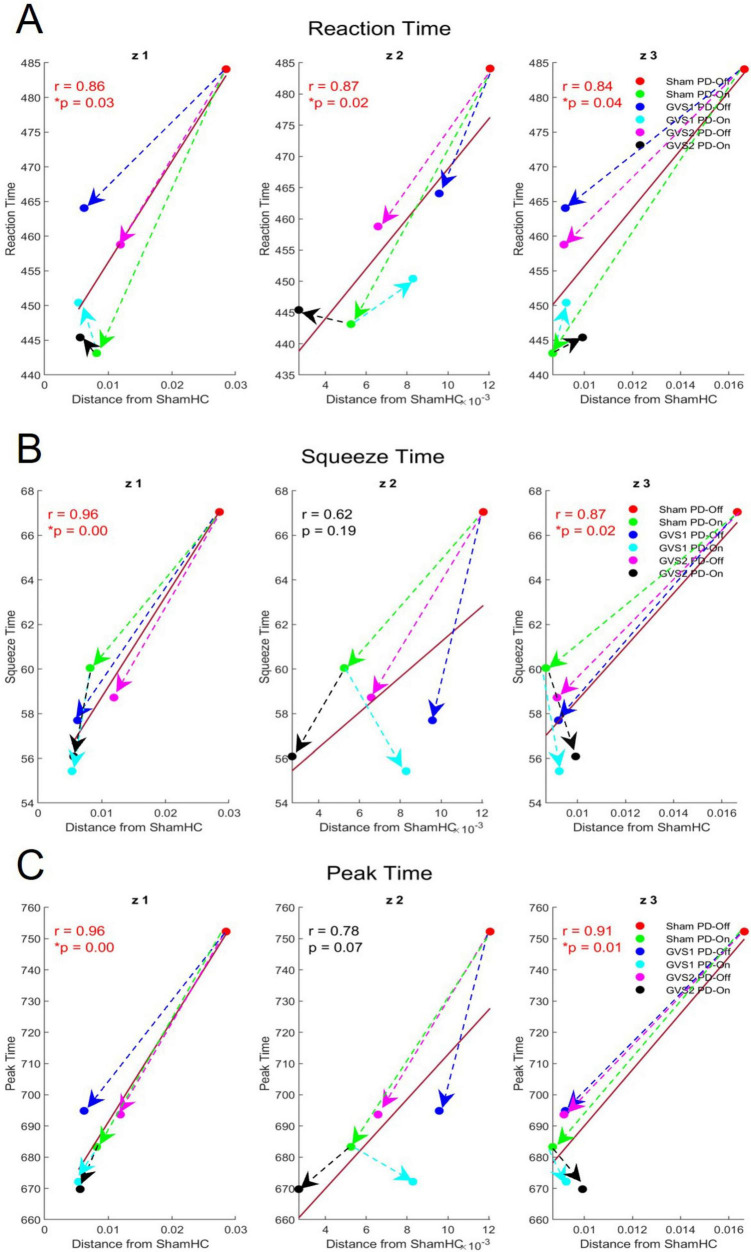
Effects of medication and stimulation on spatial contributions and motor behavior metrics across Koopman latent dimensions (*z*_1_, *z*_2_, *z*_3_). **(A–C)** Illustrate the correlations between spatial contribution distances from the HC baseline (x-axis) and behavioral metrics (y-axis) for Reaction Time, Squeeze Time, and Peak Time, respectively, across the three latent dimensions. Colored circles represent experimental groups, while arrows indicate the direction of change induced by medication and stimulation.

Figure 7 visualizes the relationship between spatial activations (measured as deviations from the HC baseline) and significant behavioral metrics (Reaction Time, Squeeze Time, and Peak Time) for each latent dimension. Across all dimensions, greater deviations from the HC baseline were associated with higher behavioral metric values (e.g., longer Reaction Time, Squeeze Time, and Peak Time). Directional arrows in Figure 7 illustrate the effects of medication and stimulation on both spatial activations and motor behavior:

• Medication Effects (Green Arrows): Medication consistently reduced spatial contribution distances and behavioral metric values, reflecting improved motor performance (shorter times) and closer alignment of spatial activations with the HC baseline.
• Stimulation Effects (Blue and Magenta Arrows): Both GVS1 and GVS2 stimulation reduced spatial contribution distances and behavioral metric values under PD-Off conditions, demonstrating their potential to enhance motor behavior.
• Combined Effects (Black and Cyan Arrows): The greatest reductions in both spatial activations and behavioral metrics were observed when medication and stimulation (e.g., GVS1 or GVS2) were combined, highlighting the cumulative benefits of these interventions.

### 3.4 LQR control for the linearized neural dynamics

The controllability analysis confirmed that the Koopman-transformed system was fully controllable, ensuring that any desired trajectory in the latent space could be tracked with appropriate control inputs. Building on this, we applied the LQR controller to align the neural dynamics of the PD-On group with the reference trajectory derived from the HC group. A representative example of the PD-On condition (Sham) system identified for LQR control design is:


$$K_{P\,D-O\,n}=\left[\begin{array}{ccc} \begin{array}{c} 0.999\\ -3\times 10^{-5}\\ 0 \end{array} & \begin{array}{c} 3\times 10^{-5}\\ 0.999\\ 0 \end{array} & \begin{array}{c} 0\\ 0\\ 0.951 \end{array} \end{array}\right]$$

**FIGURE 8:**
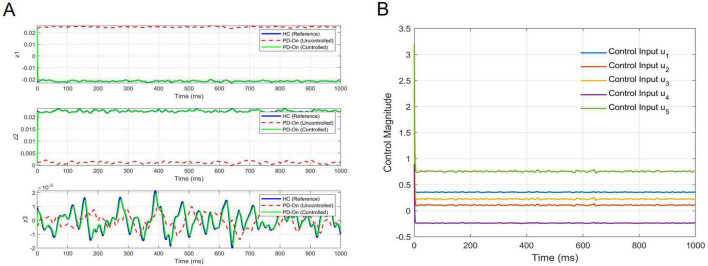
Trajectory tracking and control inputs for the LQR-controlled system. **(A)** Tracking performance: the trajectory tracking of the LQR-controlled system is shown across the three latent dimensions (*z*_1_, *z*_2_, *z*_3_) of the Koopman space. The solid blue lines represent the reference trajectories derived from the Healthy Control (HC) group, while the red dashed lines depict the uncontrolled PD-On dynamics. The green solid lines illustrate the LQR-controlled system, which aligns closely with the reference trajectories, demonstrating effective control. **(B)** Control inputs: the mapped control inputs (*u*_1_ *to* *u*_5_) are shown in the original EEG space, transformed from the Koopman latent space using the DNN decoder. These control inputs remain bounded and stable throughout the tracking period, highlighting the feasibility and robustness of this control strategy for practical implementation.

Figure 8A illustrates the trajectory tracking performance of the LQR-controlled system across the three latent dimensions (*z*_1_, *z*_2_, *z*_3_) in the Koopman space. The LQR-controlled trajectories (green) closely tracked the reference trajectories (blue), significantly reducing deviations compared to the uncontrolled PD-On trajectories (red dashed). This demonstrates that the controller effectively influenced the system dynamics in the latent space. Figure 8B depicts that the control inputs (*u*_1_ *to* *u*_5_), computed in the latent space and mapped back to the EEG domain, remained bounded and stable. Together, these results validate both the theoretical controllability and practical performance of the LQR controller in regulating neural dynamics.

## 4 Discussion

This study demonstrates the utility of a Koopman-based linearization framework for analyzing preparatory EEG dynamics in PD under GVS. While EEG is not a standard clinical tool for diagnosing or monitoring PD—as clinical evaluation remains the gold standard—it provides valuable non-invasive access to large-scale cortical dynamics., for investigating how interventions such as GVS modulate preparatory motor activity. Prior work within our research group has established the behavioral benefits of GVS, particularly using the same dataset and motor execution task analyzed in this study (Lee, 2019). High-frequency GVS (50–100 and 100–150 Hz) has been shown to enhance motor initiation, reduce movement latency, and increase response vigor in PD patients, reinforcing its potential as a neuromodulatory intervention. While previous research has largely focused on behavioral outcomes, this study extends those findings by providing a mechanistic explanation of the underlying EEG-based neural dynamics. By leveraging Koopman operator theory, we systematically examined how GVS influences cortical stability, spatial organization, and oscillatory activity during motor preparation. The deep Koopman model effectively reduced the dimensionality of EEG signals while preserving essential neural patterns, allowing for a comprehensive comparison of temporal and spatial dynamics across seven experimental groups, including HC and PD participants under different medication and stimulation conditions. These findings offer new insights into how GVS modulates motor-preparatory brain activity, bridging the gap between observed behavioral improvements and their neural underpinnings.

### 4.1 Koopman-based EEG findings and their connection to motor performance

The eigenvalue analysis quantitatively measured stability, growth, decay, and oscillatory properties of neural signals. Across all experimental groups, predominantly stable Koopman modes were observed, indicating consistent temporal dynamics in motor preparation. While numerical variations in eigenvalues were noted between groups, no statistically significant differences were found in real or complex eigenvalues. These findings suggest that while GVS and medication influence neural dynamics, the fundamental stability of motor-preparatory signals and oscillatory characteristics of dominant modes are preserved.

The spatial contribution analysis identified cortical regions that contributed to the three-dimensional Koopman latent space, linking these latent dynamics to motor preparation processes. Key findings include:

• The largest deviations from HC baseline patterns were observed in the PD-Off Sham group, indicating that cortical dynamics in untreated PD significantly diverge from normal motor preparation patterns.
• GVS1 and GVS2 reduced these deviations, with GVS1 achieving the closest alignment to HC spatial patterns, suggesting that stimulation restores cortical activity associated with motor preparation.
• The PD-On condition further reduced spatial deviations compared to PD-Off, underscoring the role of medication in partially restoring EEG-based motor-preparatory dynamics.

Importantly, correlations between EEG spatial patterns and behavioral metrics (Reaction Time, Squeeze Time, and Peak Time) confirmed the functional relevance of these EEG transformations. Closer alignment to HC EEG patterns was consistently associated with improved motor outcomes, reinforcing the hypothesis that GVS-induced EEG changes directly contribute to motor performance improvements. These findings support the hypothesis that GVS enhances motor function by restoring activity in underactive brain regions in PD. The complex spatial patterns observed in the latent space transformation (Supplementary Figure 1) likely result from the intricate projection of subcortical and cortical activity onto scalp EEG recordings. The reductions in spatial deviations from normal under GVS and medication – and the association with improved motor behavior– underscores the deep Koopman framework’s capacity to capture meaningful latent dynamics and highlights its potential for identifying therapeutic targets and developing novel strategies for intervention.

### 4.2 How this study extends prior work

While previous research primarily focused on behavioral outcomes (Lee, 2019), this study extends those findings by providing a mechanistic explanation of the EEG-based neural dynamics underlying GVS-induced motor improvements.

• Establishing a direct neurophysiological correlation of GVS-induced behavioral enhancements. Prior studies demonstrated that GVS enhances reaction time and motor execution; this study links those improvements to specific cortical activity changes during motor preparation.
• Introducing Koopman-based modeling as a novel framework for EEG analysis. Traditional EEG methods often fail to capture the full temporal structure of neural dynamics, whereas the Koopman operator approach enables linear tracking of these dynamics over time.
• Reinforcing the role of GVS in restoring cortical motor-preparatory activity. The observed GVS-driven shifts toward HC-like EEG patterns suggest that neuromodulatory stimulation can help compensate for PD-related motor deficits.

These findings highlight the potential of GVS as a neuromodulatory therapy, with specific EEG-based biomarkers that could be used to track and optimize stimulation protocols for individualized interventions.

Importantly, the observed neural effects correspond closely with previously reported behavioral improvements under GVS. Prior work using the same dataset has shown that GVS enhances reaction time, movement execution, and motor control in PD. The present findings—demonstrating the restoration of HC-like EEG spatial patterns—align with behavioral evidence that GVS reduces bradykinesia and improves movement efficiency, reinforcing the functional relevance of these EEG modulations.

Moreover, correlations between EEG spatial deviations and behavioral performance metrics (Reaction Time, Squeeze Time, and Peak Time) further support this relationship. As EEG activity under GVS became more aligned with HC patterns, participants exhibited enhanced motor performance, suggesting that restoration of cortical dynamics is functionally relevant for motor execution. These findings suggest that GVS-induced EEG changes are not merely byproducts of stimulation but actively contribute to improved motor preparation and execution.

### 4.3 Theoretical and clinical implications

This study provides important theoretical and clinical insights into the application of dynamical system modeling for neuromodulation in PD. From a dynamical systems perspective, motor control and preparatory activity can be conceptualized as trajectories within a high-dimensional state space, where the system transitions through preparatory states to achieve desired motor outcomes. The Koopman framework allows for:

• A mathematically rigorous representation of EEG signals, preserving latent motor-preparatory dynamics.
• Potential applications in real-time tracking and intervention, such as Koopman-based models can facilitate predictive modeling and closed-loop control of neural activity.
• These findings suggest that GVS, in combination with EEG-informed modeling, could serve as a personalized neuromodulation strategy to optimize treatment for PD.

In a broader context, this study addresses the challenge of interpreting high-dimensional neural activity, particularly in the context of motor impairments in PD. While methods such as Principal Component Analysis (PCA) and Independent Component Analysis (ICA) simplify neural data, they often fail to capture the temporal evolution and dynamic interactions of neural systems under non-stationary conditions. The deep Koopman-based framework combines the representational power of deep learning with the interpretability of dynamical systems, enabling robust modeling of non-linear EEG dynamics. This approach facilitates tracking and predicting neural activity over time and offers opportunities to reconfigure these dynamics through feedback and control strategies (Acharya et al., 2022), advancing therapeutic interventions for PD.

### 4.4 Methodological considerations and limitations

#### 4.4.1 EEG channel selection and dimensionality reduction

The decision to group 27 EEG electrodes into five anatomically defined ROIs was based on neurophysiological, computational, and methodological considerations, ensuring a balance between model interpretability, efficiency, and robustness. This approach aligns with the study’s goal of capturing group-level motor-preparatory dynamics, rather than focusing on isolated electrode-level variations.

• Neurophysiological Relevance: Motor-preparatory EEG activity is distributed across functionally coordinated cortical regions, rather than being confined to single electrodes (Churchland et al., 2010; Michely et al., 2015). ROI-based grouping allows for regionally specific analysis, aligning with established neurophysiological frameworks.
• Minimizing Redundancy: EEG signals exhibit spatial correlations due to volume conduction, meaning that adjacent electrodes often capture overlapping neural activity. ROI averaging reduces redundancy and enhances interpretability in spatial contribution analysis.
• Computational Efficiency and Model Stability: Directly applying the Deep Koopman model to all 27 channels would significantly increase input dimensionality, potentially requiring a higher-dimensional latent space and leading to training instability. Using five averaged signals ensures a computationally feasible representation while preserving key EEG features.

Additionally, rigorous data preprocessing, including artifact removal and 6-fold cross-validation, ensured that averaged signals remained representative and generalized well across subjects. While increasing spatial resolution by using all 27 channels could offer finer detail, preliminary tests indicated that expanding input dimensionality without adjusting the latent space structure reduced model stability.

Future work could explore adaptive channel selection strategies, sparse learning techniques, or channel-wise Koopman modeling to refine individualized cortical representations while maintaining computational feasibility.

#### 4.4.2 Identifiability and comparability of Koopman latent representations

Since each experimental group’s Koopman model was trained independently, the resulting latent spaces exist in relative coordinate systems rather than a shared absolute reference frame. This means that direct comparisons of latent feature positions across groups are not meaningful, as differences may arise from model-specific encodings rather than underlying neurophysiological distinctions.

Rather than interpreting latent space geometry in absolute terms, this study prioritizes preserving key temporal dynamics (eigenvalue analysis) and linking latent representations to EEG spatial contributions as a means of neurophysiological interpretation. The spatial contribution analysis, which anchors latent dynamics in the EEG observational space, provides a robust framework for examining group-level differences while mitigating the effects of coordinate system variability in latent representations.

Future work could explore shared embedding constraints, joint training strategies, or higher-dimensional latent representations to enhance identifiability and further refine the relationship between latent space geometry and underlying neural mechanisms.

#### 4.4.3 Validation of Koopman representations in EEG dynamics

The Deep Koopman model was validated through multiple analytical approaches to ensure that the latent space representations captured meaningful neural oscillatory dynamics relevant to motor preparation. The model’s performance was assessed using:

• Reconstruction Accuracy: High fidelity EEG reconstructions (Figure 3B) and low test errors (Supplementary Table 2) confirm that the model preserves the essential temporal and spatial structure of EEG signals, including oscillatory components.
• Temporal Dynamics via Eigenvalues: The presence of complex-conjugate eigenvalues across all groups (Figure 5) indicates that the model captures oscillatory interactions alongside non-oscillatory trends, reflecting expected neural behavior.
• Prediction Performance: The model’s ability to propagate latent EEG dynamics forward in time (Figure 4) suggests that it preserves underlying temporal evolution and transient state changes in neural activity.
• Spatial Contributions and Functional Relevance: The latent space representations align with EEG-based spatial activation patterns (Figure 7) and correlate with motor behavior metrics (Table 1), reinforcing the neurophysiological validity of the extracted dynamics.

While this study focused on real EEG data, future work could incorporate synthetic neural oscillatory systems to further characterize the model’s sensitivity to specific dynamical transitions, such as shifts between synchronous and asynchronous states. The current findings provide strong evidence that the Koopman framework effectively captures functionally relevant latent neural representations, and additional numerical validation would further enhance its applicability in modeling complex neural dynamics.

### 4.5 Future directions and conclusion

While this study provides strong evidence for EEG-based mechanisms of GVS-induced motor improvements, several areas remain for future investigation:

• Subject-specific modeling: Future work should explore individualized EEG responses to GVS, moving beyond group-level analysis to enable personalized interventions.
• Optimization of stimulation parameters: This study focused on specific GVS waveforms; future research should explore alternative stimulation paradigms to maximize therapeutic benefits.
• Validation in broader clinical settings: The Koopman framework should be tested in larger patient cohorts and different experimental conditions to establish its robustness and translational potential.

Despite these limitations, this study introduces a novel and practical approach to integrating Koopman modeling into brain stimulation research. The observed associations between EEG-derived latent dynamics and motor behavior highlight the potential of Koopman method for designing advanced stimulation strategies in PD. These findings provide a strong foundation for future research aimed at leveraging dynamical systems modeling for optimizing neuromodulatory interventions.

## Data Availability

Data supporting the findings of this study will be made available upon reasonable request to the corresponding author. The code is available in the GitHub repository at https://github.com/niktaaan/DeepKoopman_PD.
