# Supplementary material for: Koopman-based linearization of preparatory EEG dynamics in Parkinson’s disease during galvanic vestibular stimulation
Source: Front Hum Neurosci. 2025 May 14;19:1566566. doi: 10.3389/fnhum.2025.1566566 (PMC12116581; doi:10.3389/fnhum.2025.1566566)
Supplement: Supplementary file 1 [file Table_1.docx]

Supplementary Material

# Deep Neural Network Details

## Network Loss Functions

Three types of loss functions are used in training the network:

1- Reconstruction accuracy of the auto-encoder is achieved using the following loss: $\left| x-\phi^{-1}(\phi(x)) \right|$.

2- Linear dynamics are achieved using the following loss: $\left| \phi(x_{k+1})-K\phi(x_{k}) \right|$.

3- $\left| x_{k+m}-\phi^{-1}(K^{m}\phi(x_{k})) \right|$ identifies linear dynamics in the matrix K for future state prediction.

## Network Hyperparameters

*Table 1. Hyperparameters for DNN*

|  | HC | PD-Off | PD-On |
| --- | --- | --- | --- |
| Number of Hidden layers | 2 | 2 | 2 |
| Width | 70 | 40 | 70 |
| Number of Hidden layers  In auxiliary network | 2 | 1 | 1 |
| Width in auxiliary network | 30 | 145 | 160 |

# Model Performance

## Network Errors

*Table 2. Network test error for all fitted models*

| Fold | Sham-HC | Sham-PD Off | GVS1- PD Off | GVS2- PD Off | Sham- PD On | GVS1- PD On | GVS2- PD On |
| --- | --- | --- | --- | --- | --- | --- | --- |
| 1 | 4.64E-06 | 2.37E-06 | 3.19E-06 | 2.58E-06 | 4.18E-06 | 3.34E-06 | 3.86E-06 |
| 2 | 4.21E-06 | 4.41E-06 | 4.68E-06 | 5.52E-06 | 2.70E-06 | 3.56E-06 | 3.37E-06 |
| 3 | 4.47E-06 | 2.89E-06 | 1.90E-06 | 2.56E-06 | 1.97E-06 | 1.93E-06 | 2.20E-06 |
| 4 | 3.52E-06 | 2.19E-06 | 3.45E-06 | 3.43E-06 | 3.13E-06 | 3.48E-06 | 4.29E-06 |
| 5 | 4.64E-06 | 2.47E-06 | 2.17E-06 | 2.82E-06 | 2.47E-06 | 1.83E-06 | 2.33E-06 |
| 6 | 3.35E-06 | 3.40E-06 | 3.16E-06 | 3.18E-06 | 4.34E-06 | 3.40E-06 | 4.9E-06 |
| Average | 4.14E-06 | 2.96E-06 | 3.09E-06 | 3.35E-06 | 3.13E-06 | 2.92E-06 | 3.49E-06 |

*Table 3. Two factor ANOVA on PD groups dissociating the effect of medication and stimulation on network test error*

|  | Df | F | P |
| --- | --- | --- | --- |
| Medication | 1 | 0.02 | 0.87 |
| Stimulation | 2 | 0.66 | 0.52 |
| Interaction | 2 | 0.11 | 0.89 |

*Table 4. Single-factor ANOVA on Groups in Sham to dissociate the effect of health condition on network test error*

|  | df | F | P |
| --- | --- | --- | --- |
| Groups in Sham | 2 | 3.8 | **0.0464** |

*Table 5. Post-hoc paired t-test corrected for multi comparison with p<0.00166 to dissociate the effect of health condition on network test error*

| Groups in Sham | P |
| --- | --- |
| HC vs PD-off | 0.0167 |
| HC vs PD-on | 0.05 |
| PD-off vs PD-on | 0.73 |

## Prediction Error

*Table 6. Two factor ANOVA on PD groups dissociating the effect of medication and stimulation on Koopman model’s 10-step prediction error*

|  | df | F | P |
| --- | --- | --- | --- |
| Medication | 1 | 1.18 | 0.27 |
| Stimulation | 2 | 0.52 | 0.59 |
| Interaction | 2 | 0.26 | 0.77 |

*Table 7. Single-factor ANOVA on Groups in Sham to dissociate the effect of health condition on Koopman model’s 10-step prediction error*

|  | df | F | P |
| --- | --- | --- | --- |
| Groups in Sham | 2 | 2.36 | 0.104 |

*Table 8. Single-factor ANOVA to dissociate the effect of individual trials on Koopman model’s 10-step prediction error on each cross-validation fold (each trained models)*

|  | df | F | | | | | | P | | | | | |
| --- | --- | --- | --- | --- | --- | --- | --- | --- | --- | --- | --- | --- | --- |
| ShamHC | 2 | 0.25 | 0.07 | 0.07 | 0.13 | 0.7 | 0.64 | 0.98 | 0.99 | 0.99 | 0.99 | 0.69 | 0.74 |
| Sham PD-Off | 2 | 0.73 | 0.19 | 0.19 | 0.25 | 0.47 | 0.6 | 0.67 | 0.99 | 0.99 | 0.98 | 0.87 | 0.77 |
| GVS1 PD-Off | 2 | 0.45 | 1.01 | 1.05 | 0.43 | 0.51 | 0.74 | 0.88 | 0.46 | 0.43 | 0.9 | 0.84 | 0.66 |
| GVS2 PD-Off | 2 | 0.35 | 0.54 | 0.29 | 0.2 | 1 | 0.18 | 0.94 | 0.82 | 0.96 | 0.98 | 0.46 | 0.99 |
| Sham PD-On | 2 | 0.32 | 0.73 | 0.38 | 0.4 | 0.19 | 0.7 | 0.95 | 0.67 | 0.92 | 0.91 | 0.99 | 0.69 |
| GVS1 PD-On | 2 | 0.27 | 0.31 | 0.54 | 0.16 | 0.51 | 0.22 | 0.97 | 0.96 | 0.82 | 0.99 | 0.85 | 0.98 |
| GVS2 PD-On | 2 | 0.62 | 0.42 | 0.12 | 0.13 | 0.38 | 0.44 | 0.75 | 0.9 | 0.99 | 0.99 | 0.92 | 0.89 |

## Eigenvalue Analysis

*Table 9. Identified eigenvalues for all fitted models*

| Fold | Sham-HC | Sham-PD Off | GVS1-PD Off | GVS2-PD Off | Sham-PD On | GVS1-PD On | GVS2-PD On |
| --- | --- | --- | --- | --- | --- | --- | --- |
| 1 | [0.923±0.003j]  0.95 | [0.95±0.009j]  0.965 | [0.962±0.01j]  0.999 | [0.999±0.00001j]  0.999 | [0.95±0.007j]  0.999 | [0.999±0.00002j]  0.965 | [0.962±0.005j]  0.945 |
| 2 | [0.99±0.000001j]  0.94 | [0.999±0.000007j]  0.999 | [0.999±0.000003j]  0.959 | [0.999±0.000001j]  0.955 | [0.999±0.00003j]  0.951 | [0.999±0.000001j]  0.959 | [0.952±0.005j]  0.95 |
| 3 | [0.99±0.00001j]  0.99 | [0.999±0.00001j]  0.999 | [0.999±0.000007j]  0.955 | [0.999±0.000001j]  0.959 | [0.999±0.00003j]  0.954 | [0.999±0.00003j]  0.958 | [0.999±0.00007j]  0.958 |
| 4 | [0.93±0.001j]  0.94 | [0.999±0.000004j]  0.959 | [0.959±0.002j]  0.96 | [0.961±0.009j]  0.94 | [0.956±0.004j]  0.953 | [0.965±0.001j]  0.964 | [0.957±0.006j]  0.96 |
| 5 | [0.93±0.0007j]  0.95 | [0.999±0.000004j]  0.953 | [0.999±0.000005j]  0.961 | [0.961±0.01j]  0.999 | [0.953±0.001j]  0.966 | [0.966±0.002j]  0.965 | [0.959±0.006j]  0.958 |
| 6 | [0.94±0.001j]  0.93 | [0.965±0.009j]  0.944 | [0.999±0.000008j]  0.999 | [0.959±0.01j]  0.967 | [0.965±0.001j]  0.967 | [0.967±0.01j]  0.999 | [0.965±0.009j]  0.999 |

*Table 10. Two factor ANOVA for the effect of medication and stimulation on real part of complex-conjugate eigenvalue*

|  | df | F | P |
| --- | --- | --- | --- |
| Medication | 1 | 2.57 | 0.11 |
| Stimulation | 2 | 1 | 0.37 |
| Interaction | 2 | 0.28 | 0.75 |

*Table 11. Two factor ANOVA for the effect of medication and stimulation on Imaginary part of complex-conjugate eigenvalue*

|  | df | F | P |
| --- | --- | --- | --- |
| Medication | 1 | 0.006 | 0.93 |
| Stimulation | 2 | 1.8 | 0.18 |
| Interaction | 2 | 0.07 | 0.92 |

*Table 12. Two factor ANOVA for the effect of medication and stimulation on real eigenvalue*

|  | df | F | P |
| --- | --- | --- | --- |
| Medication | 1 | 0.67 | 0.41 |
| Stimulation | 2 | 0.14 | 0.86 |
| Interaction | 2 | 0.03 | 0.96 |

*Table 13. Single-factor ANOVA for the effect of disease state on real part of complex-conjugate eigenvalue*

|  | df | F | P |
| --- | --- | --- | --- |
| Groups in Sham | 2 | 2.77 | 0.094 |

*Table 14. Single-factor ANOVA for the effect of disease state on Imaginary part of complex-conjugate eigenvalue*

|  | df | F | P |
| --- | --- | --- | --- |
| Groups in Sham | 2 | 0.629 | 0.546 |

*Table 15. Single-factor ANOVA for the effect of disease state on the real eigenvalue*

|  | df | F | P |
| --- | --- | --- | --- |
| Groups in Sham | 2 | 2.598 | 0.107 |

## Analysis of Spatial Contribution

*Table 16. Two factor ANOVA for the effect of medication and stimulation on Euclidean Distances for z1*

|  | df | F | P |
| --- | --- | --- | --- |
| Medication | 1 | 4.648245 | **0.039229** |
| Stimulation | 2 | 3.17283 | 0.056238 |
| Interaction | 2 | 1.853607 | 0.174171 |

*Table 17. Two factor ANOVA for the effect of medication and stimulation on Euclidean Distances for z2*

|  | df | F | P |
| --- | --- | --- | --- |
| Medication | 1 | 3.106005 | 0.088191 |
| Stimulation | 2 | 1.464148 | 0.247332 |
| Interaction | 2 | 0.193905 | 0.82476 |

*Table 18. Two factor ANOVA for the effect of medication and stimulation on Euclidean Distances for z3*

|  | df | F | P |
| --- | --- | --- | --- |
| Medication | 1 | 1.236919 | 0.274899 |
| Stimulation | 2 | 0.724521 | 0.492842 |
| Interaction | 2 | 2.388352 | 0.109017 |

**Figure 1.** (A to C) Topographical maps of spatial activation patterns of the three Koopman latent dimensions (z1, z2, and z3) across Sham HC, Sham PD-Off Med, and GVS1 PD-On MED groups during the preparatory period.


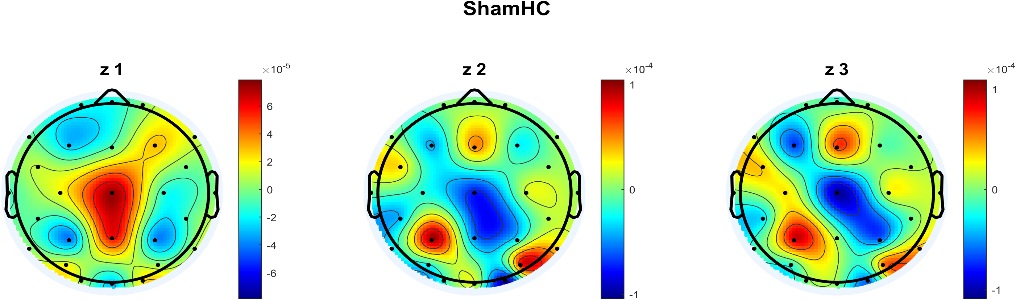

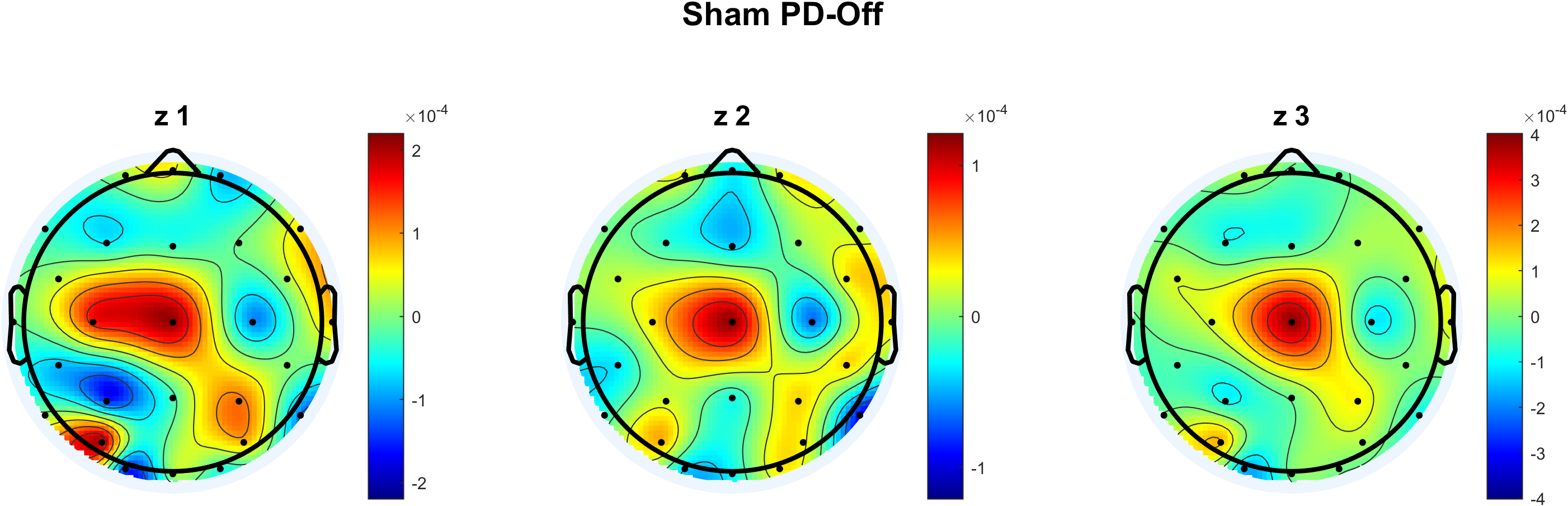

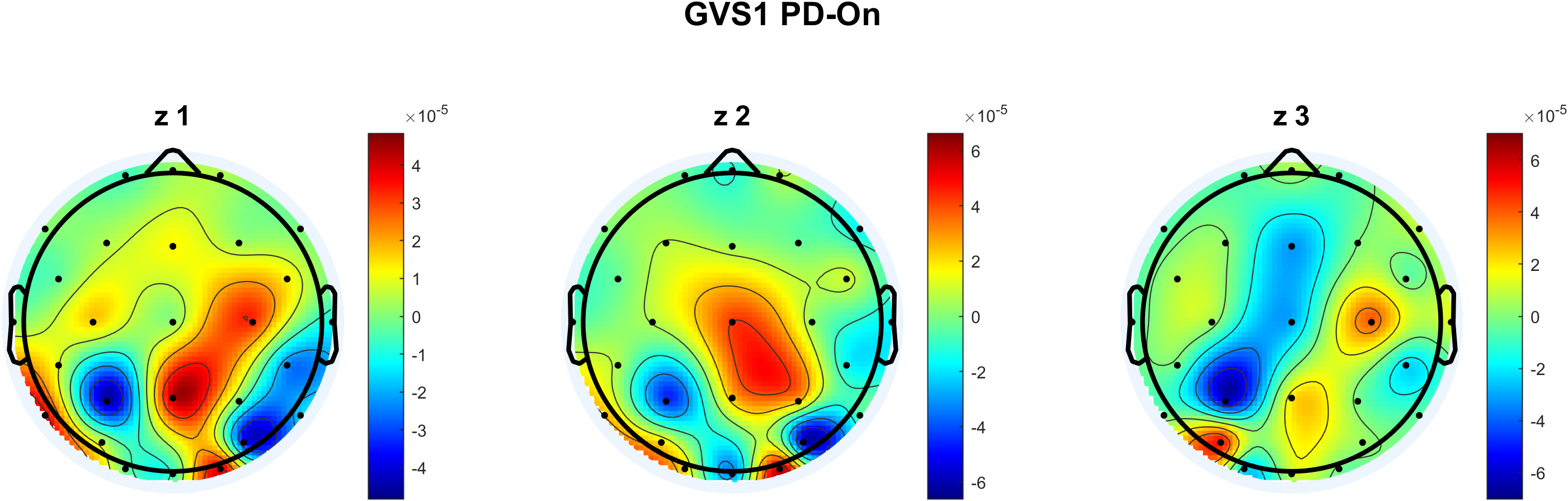


A

B

C

*Table 19. Normality Test Results for Single FactorANOVA Assumptions (p-values are reported from the Kolmogorov-Smirnov (K-S) test.)*

|  | HC | PD-Off | PD-On | Normality Assumption Met? |
| --- | --- | --- | --- | --- |
| Network Errors | 0.87 | 0.88 | 0.93 | Y |
| Prediction Errors | 0.75 | 0.75 | 0.39 | Y |
| Eigenvalue Analysis | 0.55  0.48  0.42 | 0.62  0.2  0.77 | 0.73  0.43  0.6 | Y |

*Table 20. Normality Test Results for Twe FactorANOVA Assumptions (p-values are reported from the Kolmogorov-Smirnov (K-S) test.)*

|  | PD-Off | PD-On | Normality Assumption Met? |
| --- | --- | --- | --- |
| Network Errors | 0.55 | 0.96 | Y |
| Prediction Errors | 0.82 | 0.96 | Y |
| Eigenvalue Analysis | 0.71  0.85  0.49 | 0.78  0.65  0.76 | Y |
| Spatial Contribution Analysis | 0.72 | 0.16 | Y |
